# Addressing power dynamics in community-engaged research partnerships

**DOI:** 10.1186/s41687-020-00191-z

**Published:** 2020-04-05

**Authors:** Lauri Andress, Tristen Hall, Sheila Davis, Judith Levine, Kimberly Cripps, Dominique Guinn

**Affiliations:** 1 School of Public Health, West Virginia University, Morgantown, USA; 2 Department of Family Medicine, University of Colorado, Denver, USA; 3 Boulder County Health Department, Boulder, USA; 4 Washington DC Health Department, Washington, USA; 5 South Dakota State University Extension Service, Brookings, USA; 6 Texas Southern University, Houston, USA

**Keywords:** Community engagement, Equity, Social determinants of health, Community- based participatory research, Community-academic partnerships

## Abstract

**Background:**

Successful community-engaged research depends on the quality of the collaborative partnerships between community -members and academic researchers and may take several forms depending on the purpose which dictates the degree to which power dynamics are handled within the collaborative arrangement.

**Methods:**

To understand the power dynamics and related concepts within community-engaged research arrangements, a secondary analysis of an existing qualitative data set was undertaken. Two models of community-engaged research, a review of literature, and the applied experiences of researchers familiar with community engagement practices confirmed the power dynamics concepts used to carry out the analysis of the qualitative data set according to the principles of directed content analysis. This analysis yielded quotes on power dynamics and related issues. Tools to address the power dynamics exposed by the quotes were selected using the literature and lived experience of the researchers. Finally, to ensure trustworthiness, the selected quotes on power dynamics and the recommended tools were subjected to naturalistic treatment using peer debriefings and triangulation.

**Results:**

Analysis of existing qualitative data made clear that community-engaged research between health practitioners and communities may take several forms depending on the purpose and dictate how power dynamics, including inequities, biases, discrimination, racism, rank and privilege, are handled within the collaborative arrangement. Three tools including implicit bias training, positionality, and structural competency may be used to address power dynamics and related concepts.

**Conclusion:**

Analysis of the qualitative data set highlighted the power dynamics within different community-engaged research models and the tools that may be used to address inequitable power dynamics including implicit bias training, positionality, and structural competency.

## Background

The importance of community engagement in academic research has gained in prominence and is recognized as an approach most likely to generate evidence that is trusted, meaningful, and useful to clinicians, patients, and their families when making health care decisions [1–4]. Successful community-engaged research depends on the quality of the collaborative partnerships between community -members and academic researchers and may take several forms depending on the purpose which dictates how power dynamics are handled within the collaborative arrangement [2, 5].

As a foundation for this paper on power dynamics in community engaged research, we compared two models of community engaged research including community -based participatory research (CBPR) and community-academic partnerships. While community-academic partnerships incorporate some aspects of CBPR, the form that these clinical research partnerships use to further their purpose, (i.e., translating research findings to physicians and patients) does not include all aspects of CBPR [2]. To see how purpose and form dictate concepts of power within community engaged research, Table 1 analyzes similarities and differences in CBPR and community-academic research partnerships. First, in CBPR the purpose is thought to be improving population health in a way that is compatible with and dictated by the interest, needs, and concerns of the community [6–8]. In contrast, the function of community-engaged research partnerships is to establish an effective infrastructure for translation of research out into the community through uptake by practitioners providing the discovery to patients [1]. In other words, community-academic partnerships conduct translational research with the aim of moving findings from “bench to bedside to curbside.” [1, 9, 10].

**Table 1:** Characteristics of community-based participatory research versus community academic partnerships

	Community-Based Participatory Research [6]	Community Academic Partnerships [4]
**Purpose**	Addressing structural, socio-economic, and racial/ethnic health inequities through social change by forming collaborative partnerships with communities so research reflects the priorities, identities and insights of communities.	Establishing an effective infrastructure for translation of scientific discoveries and health care recommendations into the community via adoption by practitioners for their patients
**Power**	A social determinant of health that if not addressed imperils the health status of marginalized communities.	A key factor based on rank, social status and issues of superiority between the researcher and the community that may determine the success of the engagement process.
**Key Mechanism for Change**	Empowerment or reduction of powerlessness	Engaging community members to recruit minorities into research projects
**Primary Target**	Populations conceptualized as members of community-based organizations, concerned citizens, leaders	Populations conceptualized as diverse, underrepresented patients
**Policy Implications**	Social issues, non-medical determinants of health	Healthcare policy

Community-academic partnerships conceptualize the groups they want to reach as “patients”. CBPR conceptualizes the groups of interest as “concerned citizens, community leaders, and community members” (Table 1) A key mechanism for community- academic partnerships is an agreed upon set of procedures between the health system and community that articulates why securing diverse, underrepresented patients is important [3, 4].

In community-academic partnerships issues related to power are an end to a means. The negotiation of power dynamics in community academic-partnerships is addressed solely to ensure that the community is amenable to working with the health research enterprise whose interest is in having a diverse set of medical patients for research [1–3]. Comparably, in CBPR an important mechanism is the empowerment of the community for social change [2, 6]. Power reflects how groups are included in the society in which they live which is vital to the material, psychosocial, and political empowerment that underpins social well-being and equitable health [11]. Designated as social determinants of health (SDOH) that must be addressed to improve health inequities, power, and its alternative, powerlessness, are explicit psychosocial concepts in CBPR [7, 12].

This paper uses two community -engaged models to conduct an analysis on power dynamics as expressed within an existing qualitative data set and proposes tools that may be used to address concepts associated with power dynamics in community engaged research.

## Methods

### Secondary analysis of existing data

Unless the analysis of data is conducted to answer the original proposed research questions or hypotheses and conducted by members of the research team that collected the data it is a considered secondary analyses of existing data [13]. Originally the existing qualitative data set, made up of group interviews and collected from February to April 2018, was assembled for the purpose of identifying common facilitators and barriers to community-academic partnership sustainability. Secondary data analysis is deemed an efficient way to reexamine previously collected data to generate additional insight and new knowledge [14]. Ethical questions have been raised about the distance between secondary researchers and primary data when the researchers have little experience with the data collection process and groups that provide the data [14]. To avoid this issue of distance between the researcher and the data, this study included at least one researcher involved in the collection of the primary qualitative data (TH).

Of the recommended procedures for secondary analysis of existing data we followed steps that included: (1) development of a comprehensive understanding of the data set including the population and questions under study, and method used to collect the data; and (2) development of an analytic plan and the concepts to be considered [13].

### Collection of existing data set

The collection of the existing data set occurred under a project funded from July 2017 to June 2019 by a Patient-Centered Outcomes Research Institute (PCORI) Engagement Award (Award # EAIN-6130) typically used to finance comparative effectiveness research that assesses clinical interventions in community-based settings [4]. Based on the purpose of the funding from PCORI, the qualitative data were originally collected for the purpose of identifying common facilitators and barriers to community-academic partnership sustainability.

Determined exempt from human subjects review by the Colorado Multiple Institutional Review Board (COMIRB Protocol 17–1478) the original study, in accordance with their guidelines, obtained verbal informed consent from all individual participants included in the study. The findings from the original study are being analyzed and a paper will be written and submitted for publication. Information on the original study and qualitative data set are available from the second researcher (TH) upon request.

Qualitative research is often used to expose and capture hidden experiences, voices, and views specified as key elements in theories on community engaged research and inequities in social position based on the nuanced, historical, cultural, and dynamic experiences of the groups that inhabit positions of low status and lack the power of being in the majority [15–18].

Purposeful, maximum variation sampling was used starting in December 2017 to January 2018 to target and recruit community and academic representatives from which the original qualitative data were generated [19]. To ensure heterogeneous participants, obtain a wide breadth of perspectives on the topic of sustainability, and prevent discussions from being dominated by any one perspective, the original set of participants were recruited from across the US, paying particular attention to avoid overrepresentation from university-affiliated stakeholders versus community representatives [19]. Community research liaisons from various public health and medical institutions recruited participants with community-engaged experience via telephone, email, and flyers distributed across multiple email listservs to reach a nationwide network of health practitioners and professionals. In the end 29 participants with clinical, research, and applied experiences in community -engaged research from across the U.S. made up the group in the primary study where 59% had no university affiliation, 38% identified as a patient or community stakeholder; and 76% had more than 15 years of experience in community engagement. See Table 2 for full demographic characteristics of original study participants.

**Table 2:** Demographics of participants from original study

	Total (*N* = 29) n (%)
Sector	
Academic, research or project lead	18 (62%)
Patient, community or stakeholder	11 (38%)
University Affiliation	
Yes	12 (41%)
No	17 (59%)
Years of Experience	
Less than 5 years	4 (14%)
5 to 15 years	3 (10%)
More than 15 years	22 (76%)
Region	
Midwest	3 (10%)
West	10 (34%)
South	7 (24%)
Northeast	9 (31%)
Gender	
Female	17 (59%)
Male	12 (41%)
Race/Ethnicity	
White	11 (38%)
Hispanic/Latino/a	7 (24%)
African American	6 (21%)

The twenty-nine-participants were organized into nine separate group interviews conducted by the researchers from the original project. Each of these 60- to 80-min sessions, comprised of two to five participants, convened using Zoom web-based video conferencing. Participants discussed their experiences related to sustainable or unsustainable community-academic partnerships in response to the following discussion prompt: “Focus on your most or least sustainable partnership. What was the partnership’s topic or focus? How did partnership members come together? What was your role in the partnership? In what way(s) was this partnership sustainable or unsustainable?” At the close of these sessions each member of the of the interview groups received a $100 Visa gift card. Group discussions were recorded and professionally transcribed verbatim. ATLAS.ti software (Version 8.3, ATLAS.ti Scientific Software Development, GmbH, 2018) was used to identify themes related to sustainability of community-engaged research partnerships.

### Execution of analysis of existing qualitative data

The data set used in this study consisted of nine group interviews on facilitators and barriers to sustainable community-academic partnerships. The principles of directed content analysis were used in this analysis which is appropriate if a concept or theory could benefit from further description leading to validation or an extension of that theoretical framework [20, 21]. While the original study used the transcribed group interviews to examine sustainable community-academic partnerships, in the case of this analysis, we were looking for evidence of concepts, issues, and ideas related to power dynamics in community -engaged research.

A set of codes to use in analyzing the qualitative data set was developed based on the applied experiences of the researchers (LA, TH, JL. KC, DG). Additionally, assembled during the first research project, a body of literature on CBPR and community-academic partnerships also helped to inform the development of the codes. From these resources, the researchers agreed that relevant concepts on power dynamics in community-engaged research included: stereotyping, rank, privilege, stigmatization, institutional racism, power, powerlessness, and bias [6, 7, 12, 22, 23]. Any new concepts that a researcher found were added to the list of codes.

The data set of group interviews was available in two forms. The first study generated verbatim transcripts along with versions of the transcripts loaded into qualitative data analysis software, ATLAS.ti (Version 8.3, ATLAS.ti Scientific Software Development, GmbH, 2018). The codes were applied to each form of the data set to identify quotes that related to power dynamics in community engaged research. The Atlas ti software form of the data were coded by TH. In comparison, LA reviewed and coded the verbatim transcripts. Transcripts were broken down into quote segments that were as small as possible while still remaining meaningful [24, 25]. To ensure trustworthiness, two researchers (LA and TH) compared the analysis of the two different forms of the qualitative data. Differences in coding and selected quotes were discussed and resolved between LA and TH.

### Selection of tools to address power dynamics in community-engaged research

Tools to address the power dynamics exposed by the quotes from the analysis of the existing qualitative data were chosen based on the concepts that came from the research and lived experience of the researchers (LA, SD, JL, KC, DG). Each researcher having worked at the community level for over 30 years in public health or healthcare as a practitioner, researcher or stakeholder suggested a tool that they had encountered in their work. The group came to an agreement on the tools to use after six, one-hour, Zoom based discussions. Researchers most familiar with a tool agreed to prepare a background memo of 2–3 pages (LA, SD, JL) including an example of how the tool was applied by the researcher, key ideas from the literature, and best practices on uses of the tool.

#### Validating the analysis of the existing data

To ensure trustworthiness of the selected quotes on power dynamics and the recommended tools we subjected the findings to naturalistic treatment using peer debriefings and triangulation [26–28]. The researchers used a variety of data resources including the development of background memos compiled from a review of community engaged research, analysis of the existing qualitative data set, and their lived experiences. All these findings were then exposed to inquiries where the researchers reviewed each other’s background memos, raised questions and met via Zoom conferencing to discuss and resolve issues.

## Results

The centrality of power dynamics to the success of community-engaged research was made clear by the quoted insights from the analysis of the qualitative data. Table 3 presents key quotes on power dynamics and related concepts along with three tools to help address power dynamics and achieve greater equity within collaborative engagements.

**Table 3:** Selected interview quotes on power dynamics and corresponding tools to address power differentials

Selected quotes on power	Suggested tools
*“We asked some really deep questions, and so we got to know each other. We built friendships… We all had different backgrounds, and we all represented different parts of the community. So usually these people that came together we would not—our lives would not have intersected anywhere other than here... So, I think that’s a part of the sustainability is having people that understand each other at a like real inside part of your soul kinda level.”* *-*Patient, stakeholder, or community member from western US	Implicit Bias Training is an effort to identify unconscious judgments based on ingrained stereotypes.
*“What has helped us in regard to continuing [a] relationship is some critical awareness of the forces that are making things hard for people. Concepts of shared social justice… there is a broader picture when you’re interacting with populations who have been on the social exclusion historical experience, you need to include, and respect, and instill that it’s a systemic challenge that we face together, and that we want to change and not just some sort of Band-Aid.”* -Academic/researcher from southern US	Structural Competency is recognition and understanding of systemic, institutional, and policy related barriers that cause social and health inequities.
*“The reason that we chose the researchers to work with that we did… we actually interviewed a whole bunch… One of them came in and I had a conversation with her, and I said how do you feel about partnering, and she said, ‘No.’ [Laughs] I said, ‘No?! Well, why? What is that?’ And she said, ‘Because I know nothing about American Indians. Nothing, and I really feel like you’ve got to know something about this population. I just—I have nothing to offer.’ And I said, ‘We’ll take you. Because we know nothing about research, and we’re coming in at the same place in some ways, and we’ll listen to every word you say, and you’ll listen to every word we say. You’re not coming in with preconceived notions, and we’ll have this co-learning going on all the time.”* -Patient, stakeholder, or community member from western US	Positionality is awareness of and an ongoing, internal dialogue by the researcher that examines their role in the production of knowledge and research based on a position of social status conferred on them by heritage, training, institution, gender and/or race.

### Implicit bias training

Racism embedded within the thoughts and behaviors of people over centuries translates into systems and institutions that exude individual level racism all the way to structural racism [29]. Some recognition and understanding of how social factors like racism might drive poor health physiologically and through limitations on resources and opportunities, has caused public health and medical practitioners to consider how their actions, routine practices, and the system in which they operate should incorporate pedagogy on structural racism and population health outcomes into medical training and practices [29–31].

Bias may emanate in community engagement from both ends of the community-researcher partnership based on preconceived notions and stereotyping. In Table 3 the statement used as an example for implicit bias training demonstrates the need for an effort to engage at a level that explores motivations, values, and mental frames that tend to automatically shape our reactions in a way that leads to racism and unintended stereotyping. Research suggests that provider bias plays a role in health disparities [32, 33]. Moreover, one does not have to be racist to be biased according to the science of implicit cognition which suggests that actors do not always have control over the processes of judgment, impression formation, and social perceptions [34].

Referred to as implicit bias, research in healthcare settings demonstrates that unconscious bias is negatively correlated with patient satisfaction and provider trust [32, 33]. In the case of collaborative engagement arrangements where rank and social status exist between the academic and community partners, the presence of implicit bias may undermine efforts to establish trust, communication, and equitable power. One strategy to address implicit bias is to recognize one’s own implicit biases. Researchers have designed an instrument, the Implicit Association Test, to measure implicit bias [35]. However, as a standalone strategy, awareness of implicit bias does not translate into overcoming implicit bias.

Two evidence-based practices that hold promise for overcoming implicit racial bias focus on increasing awareness and improving communication. The first intervention is based on the premise that implicit bias is like a habit that can be broken through a combination of awareness of implicit bias, concern about discrimination, and the application of strategies to reduce bias [36]. A second intervention for overcoming implicit racial bias in healthcare targets provider communication behaviors which has been shown to improve patient communication behaviors which hold the promise of reducing provider implicit bias and improving health outcomes [37, 38].

### Structural competency

Today, in the twenty-first century in the wake of the SDOH, it is no longer advisable for a practitioner to solely rely only on an understanding of a patient’s culture [39]. While cultural competency situates individual level symptoms on the bodies of marginalized groups, structural competency extends the diagnosis of individual health to populations and the contributions of institutions, systems, policies, and markets in shaping the health of those groups [40–42]. The statement in Table 3 recognizes a CBPR-like idea which is the structural barriers versus individual level issues that shape population health by controlling access to resources and opportunities for groups based on social status over a historical period of time. Referred to as “structural competency” this concept involves the recognition of structural and systemic imbalances associated with a place as risk factors that contravene the production of health and individual clinical interventions [39–41]. .Structural competency tackles power dynamics by drawing attention during a patient-physician encounter to the historical power imbalances between groups resulting in health and social inequalities. In other research to be structurally competent, researchers need an understanding of and knowledge about eight domains explained by the following assessment criteria [39].

- Financial security: resources to live comfortably
- Residence: a safe, clean, private, quiet, stable place to sleep and store possessions
- Risk environments: places to feel safe and healthy
- Food access: adequate nutrition and access to healthy food
- Social network: friends, family, or other people who help
- Legal status: legal problems
- Education: reading skills, language, and level of education
- Discrimination: self-reflection on reactions based on a stigma, biases, or negative moral judgments

Observations using a modified structural competency framework with nine domains, and four levels of proficiency was used for the analysis of a CBPR project to determine the degree to which a group of medical students exhibited an understanding of how power imbalances in institutions, systems, policies, and markets of a place shaped the health of their patients [43]. In this instance, the use of the structural competency framework to analyze the CBPR project revealed how the medical students came to an understanding of their (in)ability to see their patients only when they could experience and connect the health outcomes they saw in clinics with the place where the lives of their patients intersected with historical power dynamics that set up and maintained structural barriers.

### Positionality

The last statement used in Table 3 as an example for positionality is a positive demonstration of an exchange between the researcher and the researched community members where they both admit to power- related characteristics that could undermine the collaborative partnership if these characteristics are not recognized and explored. Because of the academic researchers role in the health system and ability to produce knowledge, CBPR imposes on the researcher a duty to recognize the power and privileges conferred upon them as trained professionals [12, 23]. These roles and the explicit acknowledgement of partiality and how knowledge is traditionally reproduced to confer privilege are referred to as positionality [44]. The notion of positionality, used in CBPR, dictates that the professional become aware of and transparent about their background and training which bestows upon them dominance, power, and superiority [12]. Developing positionality may include exercises in self-awareness and critical reflectivity or bi-directional discussions between researchers and community members where narratives are used to reveal and question the role of the researcher, the researched, and the research context and how these identities impact the ability to co-create effective research with community partners who have historical and present-day issues with power imbalances [12, 23, 45].

## Discussion

Community-engaged research partnerships open the door for communities to be authentically involved in health research. These partnerships subsequently highlight the intrinsic power dynamics that exist when academic researchers representing centers of power, privilege, and status engage with communities that have been shaped by a history of structural, socio-economic, and racial/ethnic inequities.

To acquire a better understanding of power dynamics and related concepts in community-engaged research we began with a comparison of two forms of community-engaged research including CBPR and community-academic partnerships. While community-academic partnerships incorporate some aspects of CBPR, the form that these clinical research partnerships use to further their purpose, (i.e., translating research findings to physicians and patients) does not include all aspects of CBPR [2]. In the comparison of the two community engaged models, we learned that the CBPR model was more likely to confront the power dynamics of a collaborative partnership in comparison to a community academic partnership.

Instead of addressing issues with power only if they arise, CBPR views power as a social determinant of health (SDOH) that must be distributed equitably across groups of different social status [6, 12, 22, 23]. In comparison to CBPR, we observed that community-academic partnerships view power dynamics as a concept to be addressed only if the differences in rank, privilege, and power become issues for the community [2–4]. In either model, CBPR or community-academic partnerships, trained health professionals could benefit from using a set of tools that seek to decrease the power differentials between groups by examining power dynamics and other concepts like historical and ongoing institutionalization of structural barriers to equity and power.

The quotes from the existing qualitative data set demonstrated that power dynamics were more equitable when researchers and community members adopted some of the CBPR principles such as revealing and discussing their history and arrangements that established differences in social privilege and rank. As one participant said, “*We asked some really deep questions, and so we got to know each other. …We all had different backgrounds, and we all represented different parts of the community”* (P, western U.S.). Other quotes in Table 3 reflect CBPR ideals such as the need to understand the history of a community, the population, and the context that has shaped the lives of that group. The tools presented, implicit bias training, structural competency, and positionality, aim to recognize and address power dynamics inherent in community-engaged research based on understanding differences in social status between the researcher and the researched.

## Limitations

These findings represent the analysis of an existing data set that had a limited number of participants. The extent of generalizability of the findings is unclear. Effectiveness of the identified tools to address power dynamics was not systematically studied but was instead based on the researcher’s experiences and the literature reviewed. It is possible that some other more effective tools may have been overlooked. However, the researchers involved in this study each had over 30 years of community engaged experience. We feel fairly certain that we selected effective, well known tools to address power dynamics. Recollection of community-engaged partnership experiences may have introduced recall bias to the identified themes and tools. However, this project’s focus on collecting both CBPR and community-academic partnership experiences helps reduce the chances that this recall bias distorts our findings.

## Conclusion

Community-engaged research may take several forms depending on the purpose which dictates the degree to which power dynamics are handled within the collaborative arrangement between community -members and academic researchers. Sociologically the achievement of equitable power dynamics is challenging between health researchers and populations where there are issues of rank, social status and privilege. Further, the function and formation of arrangements as either community-academic partnerships or CBPR also determine the degree to which power differentials are addressed. The present study revealed how power dynamics appear in response to different models of community-engaged research. This study highlighted CBPR as a form of community-engaged research most likely to address issues of power in community-based collaborations. Further, based up the analysis of the qualitative data set, study results indicate that community-engaged collaborations should consider incorporating principles from CBPR that that acknowledge power dynamics when academic researchers representing centers of power, privilege, and status engage with communities that have been shaped by a history of structural, socio-economic, and racial/ethnic inequities. Finally, incorporation of tools such as implicit bias training, structural competency, and positionality may be utilized to address issues with power in community engaged partnerships.

## Data Availability

The datasets used and/or analyzed during the current study are available from the corresponding author on reasonable request.

## Abbreviations

CBPR: Community-based participatory research
PCORI: Patient-Centered Outcomes Research Institute
SDOH: Social determinants of health
